# Exploring the pathophysiological mechanisms and wet biomarkers of *VPS13A* disease

**DOI:** 10.3389/fneur.2024.1482936

**Published:** 2024-11-26

**Authors:** Jingqi Lin, Hongmei Meng, Nilupaer Shafeng, Jiaai Li, Huaiyu Sun, Xi Yang, Zhiqing Chen, Shuai Hou

**Affiliations:** Department of Neurology and Neuroscience Center, The First Hospital of Jilin University, Changchun, China

**Keywords:** chorea-acanthocytosis, neuroacanthocytosis, *VPS13A* disease, *VPS13A*, chorein

## Abstract

*VPS13A* disease (also known as Chorea-Acanthocytosis, ChAc) is a representative subtype of the neuroacanthocytosis (NA) syndromes, characterized by neurodegeneration in the central nervous system and acanthocytosis in peripheral blood. It is a rare autosomal recessive genetic disorder caused by loss-of-function variants in the VPS13A gene, which is currently the only known pathogenic gene for ChAc. VPS13A protein is a member of novel bridge-like lipid transfer proteins family located at membrane contact sites, forming direct channels for lipid transport. The specific mechanism underlying how the loss of VPS13A function leads to the hematological and neurological phenotypes of the disease remains unclear. Here we present a review of recent studies on VPS13A protein and ChAc, focusing on the potential role of the VPS13A protein in pathophysiology of ChAc and also review the known and potential wet biomarkers of ChAc to enhance our comprehension of this rare disease.

## 1 Introduction

Neuroacanthocytosis (NA) is a group of rare hereditary diseases with the common clinical features of neurological degeneration and peripheral acanthocytosis, initially thought to include *VPS13A* disease (chorea-acanthocytosis, ChAc), *XK* disease (McLeod syndrome, MLS), Huntington’s disease-like 2 (HDL2), and pantothenate kinase-associated neurodegeneration (PKAN) (1). As our understanding of these diseases improves over time, PKAN is more accurately characterized as “neurodegeneration with brain iron accumulation.” Adiditionally, a meta-analysis revealed the presence of acanthocytes in 30% of individuals diagnosed with HDL2 (2), while a separate report from the same year documented the lack of acanthocytosis in all 12 HDL2 patients under examination (3). As a result, NA is now recognized as consisting of *VPS13A* disease and *XK* disease (4).

ChAc is an autosomal recessive genetic disorder caused by loss-of-function variants in the *VPS13A* gene (5, 6). It typically manifests between the ages of 20 and 40, with an average onset age of 35, rarely occurring before the age of 20 or after the age of 50 (7). The clinical presentation of ChAc is diverse, with movement disorders being the most common symptoms, primarily presenting as chorea or parkinsonism. Most patients exhibit typical oromandibular movement disorders, accompanied by lip or tongue biting and ulcers. Around 45% of patients experience seizures, which can also present as temporal lobe epilepsy without obvious motor symptoms (8), and seizures can also be the initial symptom of ChAc (9). Over half of the patients exhibit psychiatric symptoms, including obsessive-compulsive disorder, depression, anxiety, and bipolar affective disorder, which may precede the onset of motor symptoms. Some patients may also experience cognitive decline or even dementia (10). A minority of patients may have involvement of the nerves and muscles, showing decreased or absent reflexes and muscle atrophy (11). Compared to MLS, the occurrence of cardiomyopathy in ChAc is very rare (12).

MLS is an X-linked genetic disorder caused by loss-of-function variants in the *XK* gene (13). It almost exclusively affects males, with several hundred reported cases worldwide. It shares highly overlapping clinical features with ChAc, such as movement disorders, seizures, cognitive impairment, psychiatric symptoms, and neurological and muscle involvement, making it prone to misdiagnosis (12). The main distinguishing features of MLS from ChAc are the later average age of onset, a different mode of inheritance, abnormal red blood cell immunophenotype, and a higher incidence of cardiac involvement (12). The highly similar clinical features suggest a certain relationship between the functions of the two proteins, and recent researches have shown that the XK protein and VPS13A protein have an interacting relationship, which will be discussed in detail later in this article.

## 2 VPS13A, a bridge-like lipid transfer protein, is localized at membrane contact sites and forms direct channels for lipid transport

*VPS13A* (*vacuolar protein sorting 13 homolog A*) gene was named because of the high degree of homology with the yeast *Vps13* gene. It was identified by two laboratories separately in 2001 (5, 6), consisting of 73 exons spanning approximately 250 kb on chromosome 9q21. In mammals, the *VPS13* gene family contains four homologs, namely *VPS13A-D* (14). Pathogenic variants in any of these genes can lead to severe inherited neurological disorders: pathogenic variants in *VPS13A* are associated with ChAc, pathogenic variants in *VPS13B/COH1* are associated with Cohen syndrome (15, 16), pathogenic variants in *VPS13C* are associated with early-onset autosomal recessive Parkinson’s disease (17, 18), and pathogenic variants in *VPS13D* are associated with spastic ataxia, etc. (19–21). As a member of a novel bridge-like lipid transfer proteins (LTPs) superfamily, the VPS13 protein family shares similar structural features. These bridge-like LTPs have long hydrophobic grooves composed of multiple repeating units (each unit consisting of five *β*-sheets followed by a loop), which is suggested to be named the ‘repeating *β*-groove’ (RBG) domain (22, 23). LTPs containing this domain are called RBG proteins. These proteins are localized at membrane contact sites (MCSs) and bridge organelle membranes, forming direct channels for lipid large-scale and directional transport (22). They are suggested to be implicated in organelle biogenesis, membrane expansion, membrane repair, and lipid rearrangement (24).

The VPS13 protein family share analogous domain structure, and it contains several domains that are able to bind lipids and/or proteins (25). In yeast, VPS13 proteins can be recruited to MCSs between various organelles by specific adaptor proteins, and each contact site mediates different functions (26). Likewise, the VPS13A-D proteins in humans are located at different MCSs, which can explain why pathogenic variants in each gene are associated with different neurological diseases, and it also suggests the important role of membrane homeostasis in the nervous system (27).

**Figure 1:**
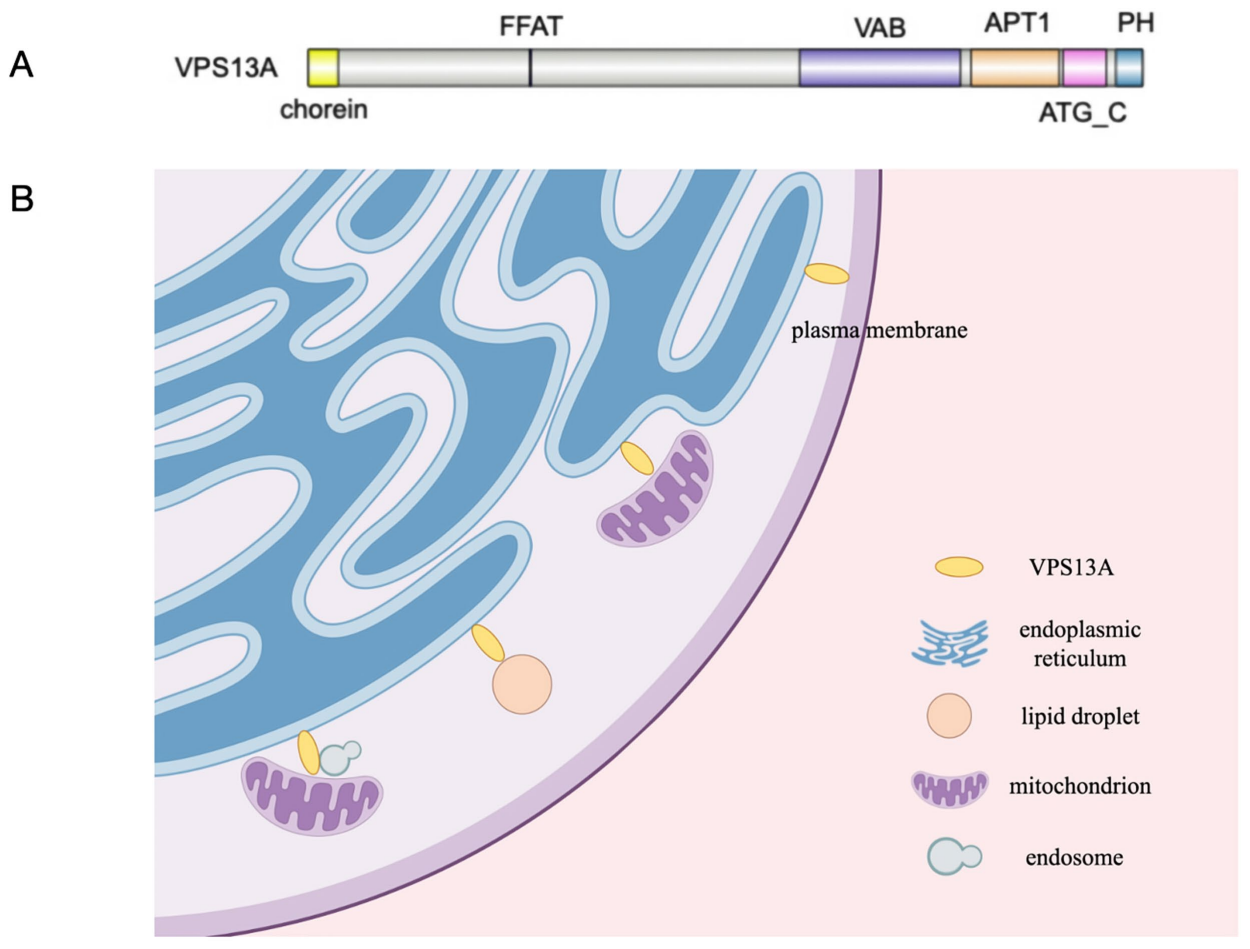
**(A)** VPS13A possesses chorein domain, VAB domain, APT1domain, ATG_C domain, PH domain and FFAT motif. **(B)** The localizations of VPS13A at various MCSs in cells according to available experimental evidences.

VPS13A possesses chorein domain, VAB domain, APT1 domain, ATG_C domain, PH domain and FFAT motif (25), and it is localized at various MCSs (Figure 1). VPS13A can be recruited to the endoplasmic reticulum (ER) through its FFAT motif by interacting with ER-resident protein VAP, and it can be recruited to mitochondria through its C-terminal domain by mediating interactions with mitochondria (28, 29). It can also be recruited to endosomes through the interactions between its VAB domain and the sorting nexin SNX5 contained in the endosomes, whereas the more C-terminal part of VPS13A directs its localization to the mitochondria, indicating that a fraction of VPS13A localizes to junctions between the ER, mitochondria, and SNX5-containing endosomes (30). VPS13A is also localized in lipid droplets and affects their movement, with an increase in the number of lipid droplets observed in mammalian cells lacking VPS13A (29). What’s more, recent research has shown a partnership between VPS13A and XK (31–34). XK belongs to the phospholipid scramblase family in the plasma membrane (PM), and it can rebalance the distribution of lipids between the leaflets when lipids are removed or inserted from donor organelles to recipient organelles. One effect of this nonspecific lipid scrambling is the translocation of phosphatidylserine (PtdSer) from the inner to the outer leaflet. Studies have suggested that VPS13A can bind to the cytoplasmic loop of XK through its C-terminal PH domain, forming the XK-VPS13A complex, which localizes at the ER-PM contact site and is regulated by XK’s internal molecular interactions (32, 33). When XK is overexpressed, it can recruit VPS13A from lipid droplets to subdomains of the endoplasmic reticulum (34). Notably, a recent study with K562 cells found that positive expression of VPS13A at ER-PM sites could be observed in differentiated cells, while it was rarely seen in undifferentiated cells, despite XK concentrations being similar before and after differentiation. It suggests that the interaction of VPS13A with XK at ER-PM contacts requires a permissive state which depends on the cell type and/or functional status (35). A recent study found that the VPS13A-XK complex is required for P2X7-mediated PtdSer exposure in T cells (36). Mutations in truncated alleles of VPS13A, discovered in *VPS13A* patients, affect the PH domain. Considering the high overlap between the clinical features of ChAc and MLS, a disruption of the XK-VPS13A complex might be the basis of both diseases and defects in PtdSer exposure may be one of the causes of neurodegenerative pathology.

## 3 Potential role of VPS13A in pathophysiology of ChAc

Loss of VPS13A function is associated with multiple phenotypes, such as disturbed autophagy (37, 38), mitochondrial dysfunction (29, 39), alteration of the actin cytoskeleton (40–43), ect. Increased Lyn kinase activity and altered signaling via PI3K have shown to be involeved in these processes (37, 42, 44). However, the specific mechanism underlying how the loss of VPS13A function leads to the blood and neurological phenotypes of the disease remains unclear. A recent study shows that brain sphingomyelin and phospholipid levels change in patients with ChAc implicate defects of lipid processing in *VPS13A* disease pathophysiology (45). As the localizations of VPS13A as LTP at MCSs has been gradually revealed, disruption of lipid exchange at one or more of these contacts is thought to be the basis for neurodegeneration and acanthocytosis in ChAc (46).

### 3.1 VPS13A in autophagy and protein homestasis

Impairment in autophagy has been shown in erythropoetic cells (37) and *VPS13A*-depleted Hela cells (38). Red blood cells of ChAc patients have impaired autophagy function during maturation process, accompanied by delayed clearance of mitochondria and lysosomes and accumulation of active Lyn (37). *VPS13A*-depleted Hela cells show aggregation of autophagic markers, decreased autophagic flux (38) and accumulation of endosomal and lysosomal markers (47). A recent study shows that rapamycin, an activator of the autophagic/lysosomal pathway, compensates the accumulation of the endo-lysosomal markers in *VPS13A* KO HeLa cells, suggesting that targeting the autophagic and lysosomal pathway modulation could be a viable therapeutic approach for ChAc. However, further validation in more complex models is necessary (48). *Drosophila* lacking the ortholog of *VPS13A* showed age associated neurodegeneration, sensitivity to proteotoxic stress and accumulation of ubiquitylated proteins in the central nervous system, partially alleviated by the overexpression of human VPS13A protein (49). Moreover, iPSC-derived medium spiny neurons (MSNs) from ChAc patients showed increased susceptibility to proteotoxic stress and endoplasmic reticulum-related UPR (39). However, these have to be further evaluated in brain tissue of ChAc patients.

### 3.2 VPS13A in mitochondria homeostasis

The absence of VPS13A in MRC-5 cells results in decreased ER-mitochondria contact sites, mitochondrial fragmentation, and reduced mitochondrial autophagy, suggesting that membrane contact microsite formation defects possibly being an indirect cause of mitochondrial dysfunction (29). Mitochondrial associated membrane sites (MAMs) is well known to be involved in the pathophysiological processes of neurodegenerative diseases, including Alzheimer’s disease, Parkinson’s disease, and others (50, 51). As one of the RBG proteins locolized at ER-mitochondria membrane contact sites, dysfunction of VPS13A also appears to have a significant impact on ChAc. The ER-mitochondria in yeast has two known lipid transport pathways: the ER-mitochondria encounter structure (ERMES) and the Vps13-Mcp1 complexes. The two pathways function in a redundant fashion and the former contributes more to lipid transport (52). Although ERMES is not identified in high eukaryotes, VPS13 is conserved thoughout eukaryotes. Introduction of *VPS13A* mutations identified in ChAc patients at cognate sites in yeast *VPS13* are specifically defective in compensating for ERMES deficiency (53). It indicated the important role of VPS13A in lipid transport at ER-mitochondria in human cells. Mitochondria impairment was also observed in iPSC-derived MSNs from ChAc patients, including reduced quantities of mitochondria, shortened mitochondrial length and hyperpolarization of membrane potential (39), suggesting that mitochondrial dysfunction may be the basis of ChAc.

Notably, phenotypic variability in *Vps13a*^−/−^ mice was observed to be modulated by the strain background (54). However, male ChAc mice were completely infertile due to significantly reduced sperm motility, possibly arising from mitochondrial abnormalities in the midpiece of the sperm, a region rich in VPS13A (55, 56). These studies also suggest the crucial role of VPS13A in maintaining mitochondrial homeostasis.

### 3.3 VPS13A and actin cytoskeleton

Cellular actin exists in the form of monomers (G-actin) and polymers (F-actin), with F-actin also known as microfilaments, which is one of the major components of the cytoskeleton. G-actin/F-actin interconvert under certain physiological conditions and maintain a balanced state within the cell, participating in a series of cellular physiological functions. Changes in the ratio between G-actin and F-actin indicate changes in cell structure, motility, and homeostasis. Studies have shown the depolymerization of cortical actin cytoskeleton in red blood cells and platelets of CHAC patients (40, 41). Increased G/F-actin ratio is also observed in patient-derived MSN of ChAc, and F-actin stabilizers can restore neuronal function (42). What’s more, human fibroblasts from chorea-acanthocytosis patients also shows significant structural disorganization of all cytoskeletal structures (43). Increased Lyn kinase activity and reduced signaling via the PI3K-Rac1-PAK pathway were reported to affect disordered actin polymerization in ChAc (42, 57).

### 3.4 Increased Lyn kinase activity and altered PI3K signaling are observed in ChAc

The abnormal accumulation of activated Lyn kinase caused by chorein protein deficiency has been proven to be responsible for part of pathological phenotypes of ChAc, and it is a potential therapeutic target to treat ChAc. Lyn is a member of the Src kinase family, and the abnormal accumulation of actived Lyn in red cells of ChAc patients can phosphorylate the anion transport protein band-3, affecting the anchoring of the RBC membrane to the cytoskeleton, leading to red blood cell deformation (44). What’s more, Src kinase inhibitor PP2 can reverse the super excitatory phenotype of iPSC-derived MSNs from ChAc patients (42). However, PP2 cannot reverse the mitochondrial and lysosomal damage observed in MSNs and midbrain/hindbrain cells induced from ChAc patient iPSCs, suggesting that the damage to mitochondria and lysosomes in central and peripheral neurons is not mediated by Lyn kinase abnormal regulation (39, 58). Compared to *Vps13a*^−/−^ mice phenocopied human ChAc, *Vps13a*^−/−^ Lyn^−/−^ mice showed normalization of RBC morphology and improvement of autophagy in basal ganglia (59). Furthermore, inhibitors of Lyn dasatinib and nilotinib have been tested in *Vps13a*^−/−^ mice phenocopied human ChAc. Nilotinib crosses the brain blood barrier (BBB), ameliorates both *Vps13a*^−/−^ hematological and neurological phenotypes, improving autophagy and preventing neuroinflammation (60). Dasatinib failed to cross the mouse BBB (60), this limitation might explain the lack of significant therapeutic effects on clinical parameters in a study involving three ChAc patients (57), but it did reduce the initially elevated Lyn kinase activity and accumulated autophagy markers in red blood cells and partially restored the actin cytoskeleton (57).

Changes in the activity of PI3K signaling pathway caused by chorein deficiency has also been shown to affect actin cytoskeleton structure and calcium homeostasis in ChAc. Foller et al. verified that the decrease of the PI3K-Rac1-PAK pathway lead to actin depolymerization and cell apoptosis in red blood cells and K562 cells of ChAc patients (42). In addition, downstream targets of PI3K include the serum and glucocorticoid inducible kinase (SGK1) (61), which regulates the activity of Na+/K + -ATPase. Consequently, chorein deficiency can also lead to impaired Na+/K + -ATPase function and decreased cellular metabolism (62). Furthermore, SGK1 can also regulate the activity of NFκB signaling, ultimately regulating the Ca2+ channel subunit ORAI1 and store-operated Ca2+ entry (SOCE) (63). SOCE is one of the important channels mediating extracellular Ca2+ influx into cells and is crucial for intracellular calcium homeostasis. Increased neurocellular apoptosis in ChAc is partly caused by reduced SOCE, and lithium can partially reverse the increased neurocellular apoptosis, suggesting its potential use in ChAc treatment (64). Based on yeast model studies, VPS13A can bind to PI3P and PI5P through its APT1 domain. The *VPS13A*-I2771R mutation found in ChAc patients leads to changes in the APT1 domain, reducing the binding of VPS13A to PI3P and PI5P and altering the localization of Vps13A in yeast (65, 66). What’s more, the APT1 domain of VPS13A binds to PI3P is regulated by Ca2+ and Mg2+. Since the intracellular Mg2+ concentration does not significantly change over time, Ca2+ controls the binding of VPS13A to PI3P. The weakened ability of mutated VPS13A protein to bind PI3P may be one of the reasons for its mislocalization and functional interruption in ChAc patient cells. It suggest that the functional connection between VPS13A and calcium signaling can be a potential intervention target for ChAc (66).

## 4 Wet biomarkers of *VPS13A* disease

Peripheral blood smear may show an increase in acanthocytes. It is generally believed that the percentage of acanthocytes in peripheral blood smear is greater than 3% can be considered positive. The percentage of acanthocytes in peripheral blood of ChAc patients is commonly between 5 and 50% (67), and the number of acanthocytes is not related to the severity of the disease. Increased acanthocytes may also appear late in the course of the disease, and the test results are greatly affected by the testing methods and operational standardization. Therefore, a negative acanthocytes result cannot rule out the diagnosis of ChAc. Storch et al. proposed that combining isotonically diluted blood samples with unfixed wet blood preparation can improve the specificity and sensitivity of the test (68). It is recommended to repeat the test multiple times and use scanning electron microscopy to observe acanthocytes to increase the detection rate. Recently, Recktenwald et al. reported the application of a device called Erysense in the detection of acanthocytes in ChAc and Huntington-like syndrome (HLS) patients. This device combines capillary chip technology with artificial neural network algorithms and can detect very subtle changes in red blood cell morphology, which may contribute to accurate identification of acanthocytes in future clinical practice (69). In addition, most ChAc patients may have a slight increase in serum creatine kinase.

Genetic testing of *VPS13A* mutations can confirm the diagnosis of ChAc. The absence of the VPS13A protein in peripheral red blood cells, as detected by Western blot testing, can also aid in the diagnosis. In a minority of cases, Western blot testing reveals normal protein levels despite genetic testing showing *VPS13A* mutations, possibly as a result of missense mutations leading to protein function loss without impacting quantity (70). Therefore, a normal chorein protein level detected by Western Blot does not rule out the diagnosis of ChAc.

Since acanthocytes are difficult to detect and identify, it is important to search for other easier and more objective ChAc biomarkers. Peikert et al. found that serum neurofilament light chain protein (sNFL) significantly increased in ChAc and MLS patients, which seems to serve as a non-specific biomarker reflecting axonal damage in the peripheral and central nervous systems. However, further evaluation is needed due to the small sample size in the study (71). Darras et al. found that the erythrocyte sedimentation rate at 2 h was significantly prolonged in ChAc and MLS patients compared to healthy controls, but further improvement is needed due to the small sample size and potential effects of age or gender on the results (72). Federti et al. found that *Vps13a*^−/−^ mice phenocopied human ChAc showed upregulation of PRX5 expression and treatment with nilotinib reduced basal ganglia oxidation and plasma levels of PRX5, suggesting that plasma PRX5 may be a biomarker for ChAc (60).

## 5 Conclusion

In this review, we summarized recent research on VPS13A protein and ChAc, exploring the potential link between the loss of VPS13A function and disease phenotypes. As the VPS13A has been identified as RBG protein in recent years, the localizations of VPS13A at various MCSs in human cells have been gradually revealed. However, the specific pathophysiological mechanisms of the disease remains unclear. Further investigation into comprehensive subcellular localization of VPS13A, the specific lipid selectivity of VPS13A, and the mechanisms regulating lipid transport might be beneficial for advancing our understanding.

Additionally, the clinical phenotype of ChAc patients caused by *VPS13A* mutations may not only be the result of interactions between genetic, protein, lipid, and other biochemical molecules. Bosman believes that the abnormal red blood cell structure in NA patients increases the sensitivity of deformed red blood cells to mechanical stress and alters their rheological properties. Potential mutations may not only affect the shape and function of red blood cells, but also make neurons in vulnerable areas of the brain more susceptible to the accompanying reduction in oxygen supply (73).

There is currently no effective treatment for the *VPS13A* disease, and symptomatic treatment measures are mainly adopted in clinical practice, including oral medication, botulinum toxin injections, deep brain stimulation, etc. (46). Due to the rarity and limited clinical sample size of ChAc, the implementation of certain clinical studies is constrained. Hence, there is a necessity to improve comprehension of the rare disease and boost detection rates.
